# Prevalence of Overweight and Obesity and Association with Risk Factors in Secondary School Children in Croatia

**DOI:** 10.3390/children11121464

**Published:** 2024-11-29

**Authors:** Antonela Matana, Helena Krajinović

**Affiliations:** The University Department of Health Studies, University of Split, Ruđera Boškovića 35, 21000 Split, Croatia; helena.krajinovic@ozs.unist.hr

**Keywords:** obesity, overweight, weight status, adolescents, prevalence, risk factors

## Abstract

Background: Childhood and adolescent overweight and obesity represent significant global health concerns. The primary objective of this study was to assess the prevalence and associations of some potential risk factors with overweight and obesity among Croatian adolescents aged 15 to 18. Methods: This cross-sectional study included 344 secondary school students from Croatia’s Mediterranean region, with data collected through an anonymous questionnaire. The questionnaire gathered sociodemographic information, adherence to the Mediterranean diet of both children and their parents, and the children’s dietary habits and physical activity levels. Results: Weight status data revealed that 2.6% of participants were underweight, 82% had normal weight, 11% were overweight, and 4.4% were classified as obese. The results indicated that boys (*p* < 0.001), children who skip breakfast (*p* = 0.031), those with less active lifestyles compared to their peers (*p* = 0.001), children whose mothers have a higher BMI (*p* < 0.001), and children from smaller families (*p* = 0.034) are at greater risk of being overweight or obese. Conclusions: This study provides valuable insights into the risk factors associated with overweight and obesity in this age group, which can support the development of targeted strategies for this population.

## 1. Introduction

Childhood and adolescent excess weight continues to be a major global health issue [1]. The estimated rates of obesity vary significantly between different countries and regions, but generally show an upward trend over time [2,3,4,5]. In 2020, the prevalence of overweight and obesity reached 21.5% in the United States and 17.6% in China, while Latin American countries reported rates between 20–25% in 2014 [3,4,5]. Within Europe, a notable geographical disparity emerged: Mediterranean countries exhibited the highest prevalence (24–37%), whereas Atlantic and Central European nations demonstrated markedly lower rates (14–21%) [2]. Recent estimates indicate that in 2022, over 390 million children and adolescents aged 5–19 were overweight [6]. The prevalence of overweight, including obesity, among children and adolescents aged 5–19 has surged from 8% in 1990 to 20% in 2022 [6]. This increase has been similar for boys and girls: in 2022, 19% of girls and 21% of boys were overweight [6]. Research indicates that Croatia has one of the highest rates of overweight and obese children in Europe, with 35% of children aged 8–9 being overweight or obese [7]. The findings indicate that the issue is more pronounced among boys, especially in the Adriatic region of Croatia [7]. Research conducted on Croatian preschool-aged children has also revealed a high rate of obesity, with 20.8% of the children being overweight or obese [8]. To the best of our knowledge, the prevalence of obesity among secondary school children in Croatia is not well-documented.

Obesity increases the risk of developing numerous chronic diseases [9]. Research has shown that, in addition to its link with conditions such as diabetes, respiratory diseases, coronary heart disease, hypertension, asthma, cancer, and others, obesity also harms psychosocial and emotional well-being and is associated with depression, anxiety, low self-esteem, and a series of emotional and behavioral disorders [9,10]. Furthermore, treating obesity in adulthood is challenging, and evidence indicates that approximately 80% of children who are overweight or obese continue to be overweight or obese as adults [11]. Therefore, preventing and treating obesity during childhood and adolescence is crucial. Several studies have reported unfavorable trends in adolescent obesity, with rates continuing to rise steadily over time [12,13,14]. These findings suggest that existing interventions are inadequate for this age group, highlighting an urgent need to develop new, targeted strategies.

The Socio-Ecological Model (SEM) provides a comprehensive framework for understanding the multifaceted determinants of childhood obesity by examining influences across various levels: individual (knowledge, attitudes, beliefs, and behaviors), interpersonal (family, peers, social networks, and associations), institutional (rules, regulations, policies, and informal structures), community (social associations, religious groups, and other networks that the child is integrated in), and policy level (legislation and policies documentation) [15,16,17,18,19]. Most studies have concentrated on specific aspects of the SEM, particularly at the individual or interpersonal levels [15].

At the individual level, lifestyle behaviors have been studied widely. Research shows that a lack of physical activity, consumption of unhealthy foods, increased sedentary behavior due to increased media use, and insufficient sleep are associated with a higher prevalence of overweight and obesity [20,21,22,23]. These unhealthy lifestyle habits have been further aggravated during the COVID-19 pandemic due to restrictions on outdoor activities, increased screen time, and disrupted sleep patterns [24]. At the interpersonal level, although several studies have identified parental education and occupation as factors associated with childhood obesity, other studies have not confirmed this association, highlighting the complex nature of family influences on weight status [25,26,27]. Within the institutional level, school-based policies on physical activity, nutrition programs, and the availability of healthy food were assessed [28,29,30,31,32,33]. Community-level research frequently focuses on neighborhood access to green space, safe recreational spaces, sidewalks, and supportive social norms for healthy behavior [34,35,36,37,38]. At the policy level, studies analyze the effects of government initiatives and legislation to restrict unhealthy foods and beverages and promote physical activity [39,40].

Despite having one of the highest rates of childhood obesity in Europe, particularly in the Adriatic region, there is limited research on obesity patterns among Croatian adolescents during the secondary school years. Additionally, Croatia’s recent socioeconomic transitions and increasing adoption of Western lifestyle patterns make it an important case study for understanding obesity trends in transitioning European societies. Therefore, this study aimed to identify the prevalence of overweight and obesity among Croatian secondary school children aged 15–18. Furthermore, we conducted a comprehensive analysis of some potential risk factors across individual (age, gender, dietary habits, physical activity) and interpersonal (parental characteristics such as education level and employment status, family meal patterns, and family structure) levels to better understand their collective influence on adolescence obesity. These findings could provide valuable insights for other countries experiencing similar dietary and lifestyle transitions, particularly in the Mediterranean regions.

## 2. Materials and Methods

### 2.1. Study Design

This cross-sectional study was conducted in the Mediterranean region of Croatia, covering the areas of Istria, Kvarner, Dalmatia, the Dubrovnik area, and the Adriatic Islands, in the period 2022–2024. A total of 344 high school students enrolled in public secondary schools, as well as their parents, were included in the study. The study was approved by the Ethics Committee of the Department of Health Studies, University of Split (Class 001-01/21-01/01, reg. no.: 2181-228-103/1-21-22). It was conducted in regulation with the latest Helsinki Declaration. The subjects provided their consent to participate by submitting the completed questionnaire.

### 2.2. Questionnaire

We sent an email invitation to all representatives of the secondary schools in the Mediterranean region of Croatia to participate in the research by completing the questionnaire designed for this study. The email included a description of the study, its purpose, and a link to the questionnaire, which was created using Google Forms. The representatives of the institutions then forwarded the questionnaire to parents via WhatsApp groups or emails. Additionally, in several institutions, paper-based surveys were distributed at the request of the institution’s representatives. For this study, the questionnaire was completed by the child’s parent (mother or father), and only one child per household was included. The questionnaire was divided into six sections. The first section focused on general information about the children, including gender, age, average grades from the previous academic year, height, and weight. The child’s parent who completed the questionnaire provided the height and weight measurements. The Body Mass Index (BMI) was calculated by dividing the child’s weight in kilograms by the square of the height in meters, allowing for the determination of BMI-for-age percentiles using the CDC growth charts [41]. Next, participants were categorized into four groups: underweight (below the 5th percentile), normal weight (5th to 85th percentile), overweight (85th to 95th percentile), and obese (95th percentile and above).

The second section consisted of questions regarding the children’s dietary habits, such as the number of main meals and snacks recorded separately for working days and off-days, the number of main meals and snacks outside the home, as well as information on eating breakfast, lunch, and dinner together as a family.

In the third part of the questionnaire, we used the KIDMED questionnaire (Mediterranean Diet Quality Index for children and adolescents) to assess adherence to the Mediterranean Diet level (MD) [42]. The KIDMED consists of 16 yes-or-no items, with 4 questions reflecting a negative association with the MD, such as consuming fast food, baked goods, sweets, and skipping breakfast, and 12 questions reflecting a positive association, including the intake of fruits, vegetables, fish, legumes, pasta or rice, nuts, oil, cereals, dairy products, and yogurt. Questions with a negative connotation were scored −1, while positive responses were scored +1, resulting in a total score ranging from −4 to 12. A KIDMED score of ≤3 indicates low adherence to the MD, a score of 4–7 indicates average adherence and a score of ≥8 indicates good adherence [42]. The questionnaire was previously adapted into Croatian and was tested for reliability and validity [43].

The next section included three questions regarding physical activity: “Does your child participate in organized physical activity (possible answers: Yes/No)?”, “How many times a week does your child do some sport, dance, or play a game in which your child is very active? (possible answers: none, 1 time, 2–3 times, 4–5 times, 6 or more times)”, “Compare your child’s level of physical activity to that of their peers” with possible answers: much less active, less active, equally active, more active, much more active.

The fifth section included questions related to the general information of both parents, including age, education level, employment status, and number of children. The family’s financial status was assessed through the question: “Do finances limit your family’s food choices?” with a yes or no response. Information on whether the child lives with both parents was also gathered. The parent completing the questionnaire reported both their own and their partner’s height and weight, which were then used to calculate BMI.

In the final section, parent’s adherence to the MD was assessed using the 14-item Mediterranean Diet Adherence Screener (MEDAS) questionnaire [44]. Each of the 14 items is scored either 1 or 0, based on whether participants follow each component of the MEDAS. The overall MEDAS score ranges from 0 to 14. Adherence to the MD was categorized as follows: weak adherence for scores ≤ 5, moderate adherence for scores 6–9, and good adherence for scores ≥ 10 [44]. The questionnaire was previously translated into Croatian and tested for reliability [45].

### 2.3. Statistical Analysis

The Kolmogorov-Smirnov test was used for normality checking. Due to the non-normal distribution of the data, continuous variables are presented as the median (interquartile range, IQR). Categorical variables are presented with frequencies (percentages). Differences in categorical variables were analyzed by using a Chi-square test, while the Kruskal-Wallis test was used for continuous variables not normally distributed. Furthermore, we performed logistic regression to assess the association of BMI categories with the odds ratios of predictors that were significant in univariate models (including gender, number of daily meals on day-offs, cereal or cereal product for breakfast, skipping breakfast, dairy product for breakfast, participation in organized physical activity, mother’s BMI, father’s BMI, mother’s education level, number of children, and physical activity compared to peers). For logistic regression analysis, we merged the two BMI categories “overweight” and “obese” into one and compared it to the “normal weight” category. We excluded underweight participants due to the small number of cases in the underweight category.

*p*-values of less than 0.05 were considered statistically significant. Statistical analysis was conducted using Statistical Package Software for Social Science, version 28 (SPSS Inc., Chicago, IL, USA).

## 3. Results

The study sample comprised 344 children aged 15 to 18, 160 boys (46.5%) and 184 girls (53.5%). The median age was 16 (IQR: 2). Data on weight status showed that 2.6% of the studied children were underweight, 82% had normal weight, 11% were overweight, and 4.4% were obese.

As shown in Table 1, there was a significant difference in weight status between boys and girls (*p* < 0.001). Overweight and obesity were more prevalent among boys in comparison to girls (17.5 vs. 5.4%, 8.1% vs. 1.1%, respectively). Age was not significantly associated with weight status (*p* = 0.863). Results from this study showed that children with normal weight had the highest average grades in the previous academic year, followed by overweight children. In contrast, obese and underweight children had lower average grades (*p* < 0.001).

Regarding the association of children’s weight status and dietary habits, the only significant association was observed for the number of daily meals on day-offs. It was shown that obese children had the highest number of daily meals on day-offs (*p* = 0.018). Results for all examined dietary habits, including the number of daily meals and snacks on working days, the number of snacks on day-offs, the number of main meals and snacks outside the home, and having breakfast, lunch, and dinner with a family, are presented in Table 2.

Although a higher prevalence of poor MD adherence was recorded among obese (46.7%) and underweight (44.4%) individuals compared to those with normal BMI (23.8%) or overweight (23.7%), no statistically significant association was observed for weight status and MD adherence (*p* = 0.173). Basic descriptive statistics of the 16 KIDMED items according to weight status are presented in Table 3. The prevalence rate of skipping breakfast was the highest for underweight children, followed by obese and overweight children, while children with normal weight skipped breakfast the least frequently (*p* = 0.05). Borderline statistical significance was observed for the consumption of cereal or cereal products for breakfast (*p* = 0.068) and consumption of dairy products for breakfast (*p* = 0.074). Underweight children consumed cereal or cereal for breakfast less frequently than obese, overweight, and normal-weight children. Obese children consume dairy products less regularly for breakfast than other weight status groups.

Results for the association of variables related to the level of physical activity and the weight status of children are presented in Table 4. Underweight and obese children were less frequently enrolled in organized physical activities compared to overweight and normal-weight children (*p* = 0.006). Furthermore, results showed that overweight and obese children were less active compared to peers (*p* < 0.001). Borderline significance was observed for the variable “How many times a week does your child do some sport, dance, or play some game in which your child is very active?” (*p* = 0.054). Normal-weight children are more frequently active more than four times a week than other weight status groups.

Results for parental socio-demographic characteristics and weight status of children are presented in Table 5. Mother’s BMI was higher for overweight and obese children (*p* < 0.001), while borderline significance was observed for father’s BMI (*p* = 0.090).

Furthermore, we performed a multivariate logistic regression for the weight status of children with eleven predictors listed in Table 6 (*p* < 0.001, Nagelkerke R^2^ = 0.435, correct prediction rate 89.6%) which were significant in the univariate model. The results of the multivariate model are presented in Table 6. Boys (*p* < 0.001), children who skip breakfast (*p* = 0.031), those with less active lifestyles compared to their peers (*p* = 0.001), children whose mothers have a higher BMI (*p* < 0.001), and children from smaller families (*p* = 0.034) are at a greater risk of being overweight or obese.

## 4. Discussion

The primary aim of this study was to determine the prevalence and factors associated with weight status in high school children from the Mediterranean region of Croatia. The results showed that 2.6% of the studied children were underweight, 82% had normal weight, 11% were overweight, and 4.4% were obese. We identified several important predictors of children’s overweight and obesity, including male gender, skipping breakfast, less physical activity compared to peers, higher BMI of mothers, and smaller number of children in a family. Results of this study also showed that academic performance is associated with the weight status of children.

Our results demonstrated that approximately 15% of high school children in Croatia were living with overweight or obesity. The results of this study indicated that the prevalence of overweight and obesity among Croatian adolescents was higher in comparison to adolescents from several other countries. For instance, in a recently performed study in Bosnia and Herzegovina, the prevalence of overweight and obesity was 10.8% [46]. A similar prevalence (11.1%) was observed in Morocco [47]. On the other hand, some countries showed a significantly higher prevalence of overweight and obesity, including Jordan (25.4%), Turkey (38.9%), Saudi Arabia (40%), and USA (46.4%) [48,49,50,51]. While still relatively high, the prevalence of excess weight in Croatian adolescents is lower than the 35% observed among children aged 8–9 [7] and 20% among preschool children in Croatia [8]. These findings align with a recently performed global meta-analysis in which a lower prevalence of obesity in adolescents was observed compared to preschool and school-age children [52]. One possible explanation for this decline in obesity prevalence during adolescence may be due to the onset of puberty. Adolescents are more vulnerable to concerns about body weight and shape than younger children, as identity development forms during this stage [53]. Body image and self-esteem are crucial in forming identity [54]. Increased academic pressure during middle and high school may also play a role in weight loss among adolescents.

Our findings confirmed that underweight and obesity were more common in boys than girls. In most high-income and upper-middle-income countries, boys are more likely to be obese than girls [55,56], and Croatia falls into this category. Several factors could justify this finding [56]. Variations in the prevalence of childhood obesity may partly be attributed to biological factors. Females generally have higher leptin levels, a hormone that reduces appetite and enhances energy expenditure [57,58]. Some evidence indicates that girls, especially in higher-income countries, may favor foods lower in calories and rich in nutrients, such as fruits and vegetables. In contrast, boys often consume more meat and calorie-dense foods [59,60,61]. Furthermore, girls tend to report higher levels of weight-related concerns compared to boys, including a greater desire to lose weight [59]. Differences could also be attributed to distinct genes contributing to differences in body composition between males and females [62]. Our research has identified skipping breakfast as one of the most significant factors associated with obesity in high-school children. This result is in accordance with previous research [63,64,65,66]. Children who skip breakfast could have a lower overall diet quality, which can contribute to excess weight [67]. The existing literature suggests that skipping breakfast is associated with diminished satiety responses throughout the day, subsequently leading to increased consumption of energy-dense, high-fat snacks [68]. Prior research examining adolescents (aged 11–16 years) in a weight loss intervention demonstrated that higher energy consumption at breakfast was inversely associated with total daily caloric intake [69]. Breakfast meals with high fiber content have been demonstrated to enhance postprandial glycemic responses, satiety and insulin sensitivity [70]. Regular breakfast consumption among children is associated with increased physical activity levels, which is postulated to be one of the mechanisms underlying the causal relationship between breakfast skipping and overweight/obesity risk in pediatric and adolescent populations [71].

In our study’s multivariate analysis, we did not identify significant associations with other dietary habits, including the number of daily meals and snacks, the number of daily meals at home, and having breakfast, lunch, or dinner together as a family. In contrast to our findings, a large cross-sectional study performed in a sample of children and adolescents from 43 countries, including Croatia, showed that a higher frequency of family meals is associated with lower odds of having excess weight and obesity [72]. The authors noted considerable variability across countries, with an overall prevalence of 50.5% of participants reporting daily family meals. In Croatia, traditional values such as family mealtimes remain widely practiced. Our study results indicate that the majority of children (ranging from 63% to 87%, depending on weight status) still share lunch or dinner with their families. This may partially explain why our study results differ from those of the previously mentioned study [72], along with the limited number of underweight and obese children included in our study. However, not all evidence, including several meta-analyses, supports the positive impact of family meals [73,74,75,76,77]. In the meta-analysis performed by Valdés et al., it was shown that, while several studies initially suggested an inverse association between the frequency of family meals and outcomes related to excess weight, this relationship lost statistical significance upon adjustment for potential confounders, including sex, age, socioeconomic status, physical activity, and diet [77]. Our study is distinct from others in that, along with child-related variables, we incorporated parental sociodemographic characteristics into the model. To further clarify this relationship, future studies with larger sample sizes and a broader range of potential confounders are required. In the existing literature, findings on the association between daily meal frequency and excess weight risk remain inconclusive. Some studies have identified a link between a higher number of daily meals and an elevated risk of excess weight [78]. Conversely, other evidence suggests a protective effect associated with increased meal frequency [79,80]. In our study, we found no evidence of association between number of daily meals and risk of overweight and obesity, which aligns with findings of a systematic review performed by Kaisari et al. [80]. These discrepancies in the literature may stem from variations in meal size and energy intake definitions across studies since questionnaire-based measures of meal frequency often lack detailed information on meal size. In the study performed by Syrad et al., the relative contributions of meal size and meal frequency were explored [81]. It was shown that a larger meal size was associated with greater weight gain, while meal frequency was not associated with weight gain, except after adjustment for meal size [81]. Therefore, future studies should analyze both meal frequencies and meal size in the same sample in the context of weight gain.

According to our results, physical activity is the most important risk factor for overweight and obesity in high school children. This is quite expected since there is a lot of evidence suggesting that a decreased level of physical activity is an important factor for the higher prevalence of childhood obesity [82]. Unfortunately, the results of our study showed that over 50% of high school students in Croatia do not participate in any organized physical activity. However, global trends in insufficient physical activity are observed. Data from 146 countries estimated that 81.0% of adolescents aged 11–17 years engage in insufficient moderate-to-vigorous physical activity [83]. In the same study, the prevalence of insufficient physical activity among adolescents in Croatia was estimated at 76.8% [83]. Considering its numerous benefits, greater efforts should be made to reverse the trend of insufficient physical activity.

The majority of the previous studies suggested that parental weight status is strongly and positively associated with children’s weight [84,85,86]. It was reported that genetic predisposition may be responsible for obesity susceptibility [87]. In our study, a higher BMI of the mother was associated with greater odds of overweight and obesity in children. Although the father’s BMI showed a borderline significance, it did not reach statistical significance in its association with the children’s weight status. However, previous studies have also shown that mothers tend to have a greater impact on children’s BMI compared to fathers [84]. The systematic review and meta-analyses performed by Heslehurst et al. revealed significantly higher odds of child obesity with increasing maternal BMI [85]. The association was most pronounced with maternal obesity, which increased the odds of child obesity by 264%, followed by maternal overweight, which raised the odds by 89% [85]. Also, another research study has shown that a mother’s BMI could contribute to 25–40% of children’s BMI [86]. There could be several explanations for these observations. Parents play a vital role in shaping the eating habits of children and adolescents, serving as influential role models. Several cross-sectional studies have demonstrated a notable and strong connection between a parent’s eating behavior and a child’s eating behavior [88]. In our recently performed study it was shown that parental Mediterranean diet adherence is, among analyzed parental predictors, the most significant predictor of children’s Mediterranean diet adherence [89]. The previous research suggests that maternal consumption of healthy food (e.g., fruit, vegetables, dairy, and cereals,) and unhealthy foods (e.g., fats, snack foods, and oils) is associated with a child’s higher intake of the same foods [90]. Mothers are usually in the role of the primary provider of food in families, including food shopping, preparation, and cooking. That could be one reason for the stronger relationship of the mother’s BMI compared to father’s BMI with excess weight in children.

In our study, parental socioeconomic status measured with indicators of education, income, and employment, was not associated with offspring overweight and obesity. In the literature, results for these associations are inconsistent. While in some European countries (Belgium, Estonia, Germany, Spain and Sweden) an inverse association between socioeconomic status and prevalence of overweight and obesity was identified, in other countries (Cyprus, Hungary, and Italy) no association was found [91]. Evidence suggests that there is an inverse relationship between socioeconomic status and childhood overweight and obesity in highly developed countries, while in areas with lower levels of development, no significant association was observed. However, there is also evidence that the relationship between socioeconomic factors and obesity varies according to the age of children. Specifically, a systematic review found that inverse association between parental socioeconomic status and offspring obesity was less prevalent among children aged 12–18 years than those aged 5–11 years, suggesting that socioeconomic differences may decrease in adolescence [92]. An abovementioned systematic review revealed that in only one of nine studies that included adolescents, the association between socioeconomic status and obesity was inverse. The results of our study support the claim that the association between socioeconomic factors and childhood obesity weakens during adolescence, likely due to increasingly similar dietary habits and levels of physical activity across different socioeconomic groups at this age.

Nevertheless, in line with our findings, previous research suggests that having more siblings is linked to a significantly lower risk of childhood obesity [93,94]. Evidence indicates that children with siblings eat healthier and watch less television [93]. Another research study indicates that boys without siblings tend to spend more time watching TV, a behavior linked to childhood adiposity [95,96]. Additionally, an increase in total screen time appears to correlate with reduced sleep duration, a factor associated with childhood obesity and unhealthy habits like skipping breakfast and consuming fast food [97]. In families with multiple children, there is often more structure around mealtimes, with a greater focus on shared family meals. Family meals are linked to healthier eating habits, such as consuming more fruits and vegetables and less fast food, reducing the risk of obesity [93]. Additionally, siblings can encourage greater physical activity and outdoor play [94]. Proposed mechanisms include peer modeling, promoting active transportation and sports participation, providing opportunities for playmates, and acting as additional caregivers [94]. This study also demonstrated that academic performance varies based on the weight status of high school students. It was shown that underweight and obese children had lower average grades compared to overweight and normal-weight children. Previous studies showed that undernutrition hampers academic achievement by stunting physical growth, hindering mental development, reducing motivation, and impairing cognitive abilities [98,99]. On the other hand, overweight and obesity can negatively impact academic performance through social factors like discrimination and stigma [100].

Several limitations of our study need to be acknowledged. The primary limitation is its cross-sectional design, which limits inference on causality. Additionally, parents completed the questionnaire for their children, which introduces the potential for misreporting the child’s nutritional quality. Also, we did not gather information on the participants’ medical conditions, which could potentially influence their weight status. Data on some other potential predictors of weight status were not collected, such as sleep habits and screen time. Additionally, the relatively small overall sample size, particularly the limited number of underweight and obese individuals, may restrict the generalizability of the findings. On the other hand, this is a comprehensive study on obesity in high school children in Croatia, which contributes to understanding of adolescent obesity in the Mediterranean region of Croatia. We examined various potential risk factors and identified several significantly associated with overweight and obesity.

In conclusion, the findings of this study revealed that a significant number of high school students in Croatia’s Mediterranean region are overweight or obese, highlighting the need for enhanced strategies to manage adolescent obesity. Findings of our study support the strategies for combating adolescent obesity which include interventions at the individual, family, and societal levels. On an individual level, encouraging children to eat breakfast regularly and engage in daily physical activity can improve their dietary and health habits. At the family level, programs involving parents, particularly those with higher BMIs, could help promote healthier family dietary and physical activity patterns. Given that smaller family size may increase the risk of obesity, educating parents on establishing healthy dietary and physical activity habits, regardless of family size, is also important. On a societal level, enhancing access to safe and affordable spaces for physical activity and promoting public campaigns on the importance of regular breakfast and physical activity can strengthen preventive measures. Interventions at all these levels, when implemented together, could have the potential to reduce the prevalence of obesity in adolescents.

## Figures and Tables

**Table 1:** Association between sociodemographic variables and the weight status of high school students.

Variables	Total Sample (n = 344)	Underweight (n = 9)	Normal BMI (n = 282)	Overweight (n = 38)	Obese (n = 15)	*p* Value
Gender						<0.001
Females	184 (53.5%)	6 (3.3%)	166 (90.2%)	10 (5.4%)	2 (1.1%)	
Males	160 (46.5%)	3 (1.9%)	116 (72.5%)	28 (17.5%)	13 (8.1%)	
Age, median (IQR)	16 (2)	16 (2)	16 (2)	16 (2)	16 (2)	0.863
Average grades in a previous academic year, median (IQR)	4.3 (1.23)	3.54 (1.38)	4.4 (1.02)	4 (1.25)	3.3 (0.8)	<0.001

**Table 2:** Association between analyzed dietary habits and weight status of high school children.

Variables	Total Sample (n = 344)	Underweight (n = 9)	Normal BMI (n = 282)	Overweight (n = 38)	Obese (n = 15)	*p* Value
Number of daily meals on working days, median (IQR)	3 (0)	3 (0.5)	3 (0)	3 (0)	3 (1)	0.453
Number of daily meals on day-offs, median (IQR)	3 (0)	3 (0)	3 (0)	3 (0)	3 (1)	0.018
Number of snacks on working days, median (IQR)	2 (0)	1.5 (1.5)	2 (0)	2 (1)	2 (1)	0.117
Number of snacks on day-offs, median (IQR)	2 (1)	2 (1)	2 (1)	2 (1)	2 (1.5)	0.581
Number of daily meals outside the home, median (IQR)	1 (0)	1 (1)	1 (0)	1 (1)	1 (0)	0.996
Number of snacks outside the home, median (IQR)	1 (1)	1 (1)	1 (1)	1 81)	1 (1)	0.308
Having breakfast together as a family, n (%)	135	3 (33.3%)	116 (41.4%)	13 (34.2%)	3 (20%)	0.357
Having lunch together as a family, n (%)	281	7 (77.8%)	230 (81.6%)	33 (86.8%)	11 (73.3%)	0.741
Having dinner together as a family, n (%)	251	7 (77.8%)	207 (73.9%)	24 (63.2%)	13 (86.7%)	0.353

**Table 3:** Association between KIDMED items and weight status of high school children.

	Total Sample (n = 344)	Underweight (n = 9)	Normal BMI (n = 282)	Overweight (n = 38)	Obese (n = 15)	*p* Value
KIDMED index score, median (IQR)	5 (4)	4 (4)	5 (3)	5.5 (4)	4 (6)	*p* = 0.175
KIDMED index score, n (%)						
Poor	87 (25.3%)	4 (44.4%)	67 (23.8%)	9 (23.7%)	7 (46.7%)	*p* = 0.173
Average	198 (57.6%)	5 (55.6%)	168 (59.6%)	20 (52.6%)	5 (55.6%)	
Good	59 (17.2%)	0 (0%)	47 (16.7%)	9 (23.7%)	3 (20%)	
KIDMED items, n (%)						
Fruit or fruit juice daily	282 (82%)	7 (77.8%)	233 (82.6%)	30 (78.9%)	12 (80%)	*p* = 0.829
Second serving of fruit daily	144 (41.9%)	4 (44.4%)	115 (40.8%)	19 (50%)	6 (40%)	*p* = 0.735
Fresh or cooked vegetables daily	229 (66.6%)	4 (44.4%)	192 (68.1%)	26 (68.4%)	7 (46.7%)	*p* = 0.173
Fresh or cooked vegetables > 1/day	71 (20.6%)	1 (11.1%)	58 (20.6%)	8 (21.1%)	4 (26.7%)	*p* = 0.843
Regular fish consumption (at least 2–3/week)	76 (22.1%)	3 (33.3%)	57 (20.2%)	12 (31.6%)	4 (26.7%)	*p* = 0.259
>1/week fast-food (hamburger) restaurant	40 (11.6%)	0 (0%)	36 (12.8%)	1 (2.6%)	3 (20%)	*p* = 0.118
Pulses > 1/week	195 (56.7%)	4 (44.4%)	159 (56.4%)	22 (57.9%)	10 (66.7%)	*p* = 0.762
Pasta or rice almost daily (≥5 days/week)	76 (22.1%)	2 (22.2%)	65 (23%)	6 (15.8%)	3 (20%)	*p* = 0.802
Cereal or cereal product for breakfast	200 (58.1%)	2 (22.2%)	169 (59.9%)	23 (60.5%)	6 (40%)	*p* = 0.068
Regular nut consumption (at least 2–3/week)	149 (43.3%)	3 (33.3%)	123 (43.6%)	18 (47.4%)	5 (33.3%)	*p* = 0.766
Use of olive oil at home	307 (89.2%)	8 (88.9%)	253 (89.7%)	35 (92.1%)	11 (73.3%)	*p* = 0.222
No breakfast	72 (20.9%)	4 (44.4%)	50 (17.7%)	12 (31.6%)	6 (40%)	*p* = 0.050
Dairy product for breakfast	292 (84.9%)	7 (77.8%)	245 (86.9%)	30 (78.9%)	10 (66.7%)	*p* = 0.074
Commercially baked goods or pastries for breakfast	176 (51.2%)	4 (44.4%)	145 (51.4%)	17 (44.7%)	10 (66.7%)	*p* = 0.539
Two yogurts and/or 40 g cheese daily	156 (45.3%)	3 (33.3%)	132 (46.8%)	14 (36.8%)	7 (46.7%)	*p* = 0.615
Sweets and candy several times a day	91 (26.5%)	2 (22.5%)	74 (26.2%)	9 (23.7%)	6 (40%)	*p* = 0.653

**Table 4:** Results for the association of variables related to the level of physical activity and the weight status of children.

	Total Sample (n = 344)	Underweight (n = 9)	Normal BMI (n = 282)	Overweight (n = 38)	Obese (n = 15)	*p* Value
Does your child participate in organized physical activity?	161 (47%)	1 (11.1%)	145 (51%)	13 (34%)	4 (26.7%)	0.006
How many times a week does your child do some sport, dance or play some game in which your child very active?						0.054
None	56 (16.2%)	3 (33.3%)	39 (14.1%)	9 (24.3%)	5 (33.3%)	
1 time	54 (15.7%)	1 (11.1%)	39 (14.1%)	9 (24.3%)	5 (33.3%)	
2–3 times	103 (29.9%)	4 (44.4%)	86 (31.2%)	10 (27%)	3 (20%)	
4–5 times	72 (20.9%)	0 (0%)	66 (23.9%)	4 (10.8%)	2 (13.3%)	
6 or more times	52 (15.1%)	1 (11.1%)	46 (16.7%)	5 (13.5%)	0 (0%)	
Compare your child’s level of physical activity to that of their peers:						<0.001
Much less active	10 (2.9%)	0 (0%)	5 (1.8%)	3 (7.9%)	2 (13.3%)	
Less active	58 (16.9%)	1 (11.1%)	41 (14.5%)	12 (31.6%)	4 (26.7%)	
Equally active	119 (34.6%)	4 (44.4%)	93 (33%)	15 (39.5%)	7 (46.7%)	
More active	89 (25.9%)	3 (33.3%)	81 (28.7%)	4 (10.5%)	1 (6.7%)	
Much more active	63 (18.3%)	0 (0%)	59 (20.9%)	4 (10.5%)	0 (0%)	

**Table 5:** Results for parental socio-demographic characteristics and weight status of children.

	Total Sample (n = 344)	Underweight (n = 9)	Normal BMI (n = 282)	Overweight (n = 38)	Obese (n = 15)	*p* Value
Number of children, median (IQR)	2 (1)	3 (2)	2 (1)	2 (0)	3 (2)	0.024
Mother’s BMI, median (IQR)	23.5 (4.41)	22.5 83.16)	23 (3.88)	25.2 (5.04)	27.8 (3.6)	<0.001
Father’s BMI, median (IQR)	27.4 (4.03)	24.9 (4.6)	27.2 (4)	27.7 (3.7)	30.7 (5.9)	0.090
Mother’s age, median (IQR)	45 (8)	47 (12)	45 (8)	43.5 (7.8)	48 (11)	0.687
Father’s age, median (IQR)	47 (9)	47 (13)	47 (8)	48.5 (12.8)	49 (15)	0.964
Mother’s Educational Level						0.059
Primary or high school	209 (61.5%)	7 (77.8%)	170 (81.2%)	19 (50%)	13 (86.7%)	
Bachelor degree	18 (5.3%)	1 (11.1%)	13 (4.7%)	3 (7.9%)	1 (6.7%)	
Master or PhD	113 (33.2%)	1 (11.1%)	95 (34.2%)	16 (42.1%)	1 (6.7%)	
Father’s Educational Level						0.135
Primary or high school	222 (67.9%)	7 (77.8%)	178 (66.9%)	22 (59.5%)	15 (100%)	
Bachelor degree	26 (8%)	0 (0%)	22 (8.3%)	4 (10.8%)	0 (0%)	
Master or PhD	79 (24.2%)	2 (22.2%)	66 (24.8%)	11 (29.7%)	0 (0%)	
Living with Both Parents, n (%)						0.595
Yes	292	6 (66.7%)	238 (84.4%)	34 (89.5%)	14 (93.3%)	
No	46	3 (33.3%)	38 (13.5%)	4 (10.5%)	1 (6.7%)	
Do Finances Limit Your Family in Food Choices?, n (%)						0.560
Yes	99 (29.3%)	3 (33.3%)	77 (27.8%)	14 (37.8%)	5 (33.3%)	
No	239 (70.7%)	6 (66.7%)	200 (72.2%)	23 (62.2%)	10 (66.7%)	
Parental employment status, n (%)						0.155
both parents employed	252 (75.4%)	4 (44.4%)	205 (75.4%)	31 (81.6%)	12 (80%)	
at least one parent is unemployed	82 (24.6%)	5 (55.6%)	67 (24.6%)	7 (18.4%)	3 (20%)	
MEDAS categories, n (%)						0.328
low	82 (23.8%)	4 (44.4%)	66 (23.4%)	7 (18.4%)	5 (33.3%)	
moderate	241 (70.8%)	5 (55.6%)	200 (70.9%)	26 (68.4%)	10 (66.7%)	
high	21 (6.1%)	0 (0%)	16 (5.7%)	5 (13.2%)	0 (0%)	

**Table 6:** Results of a multivariate logistic regression for the weight status of children.

Variable	OR	95% C.I.	*p*-Value
Gender (females)	0.151	(0.064, 0.353)	<0.001
Number of daily meals on day-offs	0.465	(0.174, 1.245)	0.128
Cereal or cereal product for breakfast (no)	0.736	(0.325, 1.67)	0.461
No breakfast (no)	2.77	(1.095, 7.002)	0.031
Dairy product for breakfast (no)	1.21	0.45, 3.28	0.71
Does your child participate in organized physical activity (no)	0.678	(0.271, 1.69)	0.405
Mother’s BMI	1.26	(1.12, 1.42)	<0.001
Father’s BMI	1.01	(0.91, 1.13)	0.822
Mother’s education level	0.691	0.29, 1.65	0.554
Primary or Secondary School	1.33	0.28, 6.21	0.404
Bachelor	0.61	0.38, 0.96	0.719
Master or PhD	-	-	-
Number of children	0.607	(0.382, 0.964)	0.034
Compare your child’s level of physical activity to that of their peers:			0.001
Much less active	49.77	4.8, 513.6	0.001
Less active	8.5	1.67, 43.03	0.01
Equally active	8.7	2.08, 37.05	0.003
More active	1.39	0.28, 6.89	0.691
Much more active	-	-	-

## Data Availability

Raw data can be found at the corresponding author via e-mail: antmatana@ozs.unist.hr.
